# Case of Periodical Insanity, Corresponding with the Phases of the Moon, and Connected with Biliary Derangement

**Published:** 1830-10-01

**Authors:** 


### XXII.

Case of Mental Derangement; occurring in Periodical Accessions corresponding with the Phases of the Moon, and apparently connected with Hepatic Disorder. Reported by J. Ferguson, M.D. Assist. Surgeon, 27th Regt.*

Private Peter Keinan, 27th Regt. æt. 26, has been only three years in the West Indies, and remarked by his comrades for being wild and silly in his general behaviour. He was on the hospital guard at Grenada on the 3d of March, 1829, when Major-General Sir J. C. visited the hospital, and was scarcely relieved off sentry, when he presented himself to the General's notice, and behaved in the most extravagant manner, proclaiming himself a deserter from the 58th Regt. He was deprived of his arms, and put in confinement; where he became quite outrageous, singing and cursing, at intervals, tearing his clothes and breaking every thing he could lay hold of. He slept little, and talked much, returning answers to his own questions; he continued in this state till the 16th, when he had a lucid interval, which lasted four days, and then he relapsed into his former state — dancing, singing, and talking to himself. He tore whatever clothes were given him, and lay naked on the floor of his cell, appearing quite insensible to cold. His appetite was so ravenous, that he once caught a fowl, and eat all but the skin and feathers. His *stools* were *black* and *scanty*. The first efforts were therefore directed to restore the healthy state of the *biliary secretion;* but it was necessary to select the lucid periods for this purpose, as he smeared his person with his feces, if medicine was given in his outrageous state. He had *two lucid intervals* every month, and the change from the insane to the sane state corresponded exactly with the *lunar changes*—a circumstance which was only discovered when the case was closed, by comparing the *register* with the *almanack.* His head was kept close shaved, and shower-baths given once or twice a day with good effect. He was also bled once, when outrageous, without producing any mitigation of symptoms—his pulse was generally at 50—skin cool, emitting that fetor so peculiar to the insane—tongue clean—made no complaint of his head. He took a five grain calomel pill three times a day, during three lucid periods, each of four or five days duration—having previously used blue pill in the same proportion, without effect—and on the 11th of June his mouth became, for the first time, affected by the mercury. He was quite coherent and melancholy—sobbed bitterly all day—his pulse quickened, and his feces assumed a more natural appearance; from this period was dated his recovery, as he had only two slight accessions afterwards, and is now at his duty in robust health, having had no attack for the last eleven months, and the Adjutant remarks that he is much quicker at his drill than before his illness.

*Barbados, June 20th,* 1830.

* Regimental Hospital, 27th Regt.

